# The role of sphingolipids in heart failure

**DOI:** 10.1093/ehjopen/oeaf035

**Published:** 2025-05-02

**Authors:** Rana Hamouche, Scott A Summers, William L Holland, Sutip Navankasattusas, Stavros G Drakos, Eleni Tseliou

**Affiliations:** Nora Eccles Harrison Cardiovascular Research and Training Institute, University of Utah, Salt Lake City, UT 84112, USA; Diabetes and Metabolism Research Center, Department of Nutrition and Integrative Physiology, University of Utah, Salt Lake City, UT 84112, USA; Diabetes and Metabolism Research Center, Department of Nutrition and Integrative Physiology, University of Utah, Salt Lake City, UT 84112, USA; Nora Eccles Harrison Cardiovascular Research and Training Institute, University of Utah, Salt Lake City, UT 84112, USA; Nora Eccles Harrison Cardiovascular Research and Training Institute, University of Utah, Salt Lake City, UT 84112, USA; Division of Cardiovascular Medicine, Department of Internal Medicine, University of Utah Health & School of Medicine, Salt Lake City, UT 84132, USA; Nora Eccles Harrison Cardiovascular Research and Training Institute, University of Utah, Salt Lake City, UT 84112, USA; Division of Cardiovascular Medicine, Department of Internal Medicine, University of Utah Health & School of Medicine, Salt Lake City, UT 84132, USA

**Keywords:** Heart failure, Ceramides, HFrEF, HFpEF, Sphingolipids

## Abstract

Advanced heart failure (HF) is characterized by changes in the structure, function, and metabolism of cardiac muscle. As the disease progresses, cardiomyocytes shift their ATP production from fatty acid oxidation to glycolysis. This shift results in an accumulation of lipid metabolites, particularly sphingolipids, which can disrupt normal cellular function and contribute to cardiac dysfunction. In animal models of obesity, accumulation of toxic sphingolipid metabolites in the heart has been described as cardiac lipotoxicity. In humans, HF is classified into two groups based on ejection fraction (EF): HF with reduced EF of less than 40% (HFrEF) and HF with preserved EF of greater than 50% (HFpEF). Despite shared risk factors and comorbidities, the structural and cellular differences between HFrEF and HFpEF distinguish them as separate conditions. Ceramides (Cer), a type of sphingolipid, have gained significant attention for their involvement in the development and prognosis of atherosclerotic disease and myocardial infarction, while sphingosine-1-phosphate, a downstream product of Cer, has shown cardioprotective properties. The aim of this review is to describe the role of sphingolipids in HF with reduced and preserved EF. By understanding the role of sphingolipids through animal and human studies, this review aims to pave the way for developing strategies that target abnormal signalling pathways in the failing heart, ultimately bridging the gap between scientific research and clinical applications.

## Introduction

The heart relies on a significant amount of ATP to carry out its contractile function. Its main energy source is fatty acids (FAs), followed by carbohydrates, lactate, and other substances like ketones and branched-chain amino acids.^1^ These energy substrates need to be continuously supplied to the cardiomyocytes through the bloodstream since the heart has a limited ability to store them.^2^ However, in cases of heart failure (HF), the heart’s metabolism undergoes significant changes. The failing heart shifts away from relying on FA oxidation and instead prioritizes glycolysis and ketone oxidation as the primary source of ATP production in cardiomyocytes.^3^ Multiple factors contribute to the decrease in FA oxidation, including mitochondrial dysfunction^4^ and alterations in gene expression.^5^ The accumulation of lipid intermediates will then disrupt normal cellular processes, leading to oxidative stress, mitochondrial dysfunction, impaired calcium signalling, inflammation, and cell death. In this review, we provide an overview of sphingolipid metabolism in the healthy heart and the alterations in cardiac and systemic sphingolipid metabolism both in HF with preserved ejection fraction (HFpEF) and HF with reduced ejection fraction (HFrEF). We also discuss human and animal models of sphingolipid metabolism-related diseases.

## Sphingolipid metabolism in the healthy heart and in heart failure

Sphingolipids are a class of bioactive lipids that play a role in cellular signalling and apoptosis. Of this lipid class, ceramides (Cer) have received the most significant attention due to their role in the pathophysiology of multiple diseases and their potential role as biomarkers and predictors of cardiovascular and metabolic diseases.^6–8^ Sphingolipidoses or lysosomal storage diseases are a group of inherited metabolic diseases with a frequency of 1 in 5000 births with deficiencies in enzymes involved in the sphingolipid pathway.^9^ Affected individuals develop mainly degenerative neurological symptoms and disorders related to other systems including the gastrointestinal, cardiovascular, musculoskeletal, and respiratory.^10^ Cer have been linked to metabolic and neurodegenerative diseases such as obesity, diabetes mellitus,^11–13^ Alzheimer’s disease,^14,15^ and Parkinson’s disease.^16,17^ However, the exact role of Cer and their mechanism of action in cardiomyocytes are not completely understood. *Table 1* summarizes the cardiac phenotypes seen in sphingolipid-related diseases. Although some sphingolipid diseases do not manifest a cardiac phenotype, the absence of such a phenotype does not preclude the possibility that it could have developed if patients had lived longer. Indeed, the lifespan of patients with sphingolipidoses typically ranges from 1 to 5 years, depending on the type of the disease,^18^ which may not provide adequate time for the deleterious effects of toxic product accumulation in cardiomyocytes to impact cardiac function.

**Table 1:** Sphingolipid-related diseases and their cardiac phenotype

Disease	Gene	Encoded protein	Cardiac phenotype	Reference
Fabry disease	GLA	α-Galactosidase A	Hypertrophic cardiomyopathy, left ventricular hypertrophy, diastolic dysfunction, arrhythmias, valvular involvement	^91–93^
Farber disease	ASAH1	Acid ceramidase	Valvular heart disease, granulomatous lesions on heart valves	^94^
Gaucher disease	GBA	Glucocerebrosidase	Pericardial effusion, cardiac hypertrophy, valvular involvement (mitral and aortic valve calcification)	^95,96^
GM1 gangliosidoses	GLB1	β-Galactosidase	Cardiomyopathy	^97^
Krabbe disease	GALC	Galactocerebrosidase	No cardiac phenotype	
Metachromatic leukodystrophy	ARSA	Arylsulfatase A	Hypertension secondary to end-stage renal disease	^98^
Niemann–Pick disease	SMPD1	Sphingomyelinase	Endomyocardial fibrosis, valvular heart disease, coronary artery disease	^99^
Pompe disease	GAA	Acid α-glucosidase	Hypertrophic cardiomyopathy	^100,101^
Sandhoff disease (GM2 gangliosidoses type II)	HEXB	Beta-hexosaminidase	Valvular heart disease, metabolic cardiomyopathy	^102,103^
Tay–Sachs disease (GM2 gangliosidoses type I)	HEXA	Hexosaminidase A	Rarely cardiac phenotype present	^104^

Our understanding of cardiac sphingolipid metabolism mainly stems from animal studies, primarily rodent models with genetic or diet-induced metabolic alterations and human studies of substrate utilization in the adult heart. The entry of FA into cardiomyocytes depends mainly on circulating non-esterified FAs and esterified FAs bound to lipoproteins.^19^ Transporters such as FA transport protein (FATP), FA translocase (CD36), and plasma membrane FA binding protein (FABP_pm_) facilitate the entry of FA into cardiomyocytes.^20^ In healthy cardiomyocytes, FA follows three pathways: beta-oxidation for ATP production (the most active pathway), production of triglycerides (TAG), or involvement in the sphingolipid pathway. It has been demonstrated that sphingolipids are widely distributed throughout the cardiac tissue and are involved in various cardiac functions including contractility, myocardial hypertrophy, and fibrosis.^21–23^

Despite their distinct pathophysiological characteristics, HFrEF and HFpEF share similarities, including metabolic inflexibility in cardiomyocytes. Heart failure with reduced ejection fraction often develops after an ischaemic insult or dilated cardiomyopathy while HFpEF is associated with factors such as female sex, obesity, diabetes mellitus, and hypertension.^24,25^ However, both conditions involve alterations in ATP production by cardiomyocytes accompanied by reduced FA entry and oxidation.^26^ Both animal and human studies have identified a shift in substrate utilization towards glucose and lactate and away from FA.^1,27,28^ The mechanisms underlying this change in substrate preference are poorly understood and might differ between early- and end-stage HF. Increased circulating levels of acyl-carnitines, intermediates of FA oxidation, in HF patients were initially interpreted as a sign of impaired cardiac FA oxidation,^29,30^ but subsequent studies indicated that myocardial levels of FA oxidation intermediates are instead reduced in HF and that the failing heart does not release acyl-carnitines. It must be noted that not all studies in humans were consistent, with some reporting unchanged or even increased FA uptake and oxidation.^31,32^ This inconsistency may be attributed to co-existing comorbidities, such as obesity and diabetes, which are associated with a shift in substrate towards FA oxidation. Discrepancies related to the stage and cause of left ventricular dysfunction were also noted in animal models.^33,34^ Nonetheless, FA remain the primary ATP source for the failing heart.

## Sphingolipid biosynthesis

**Figure 1:**
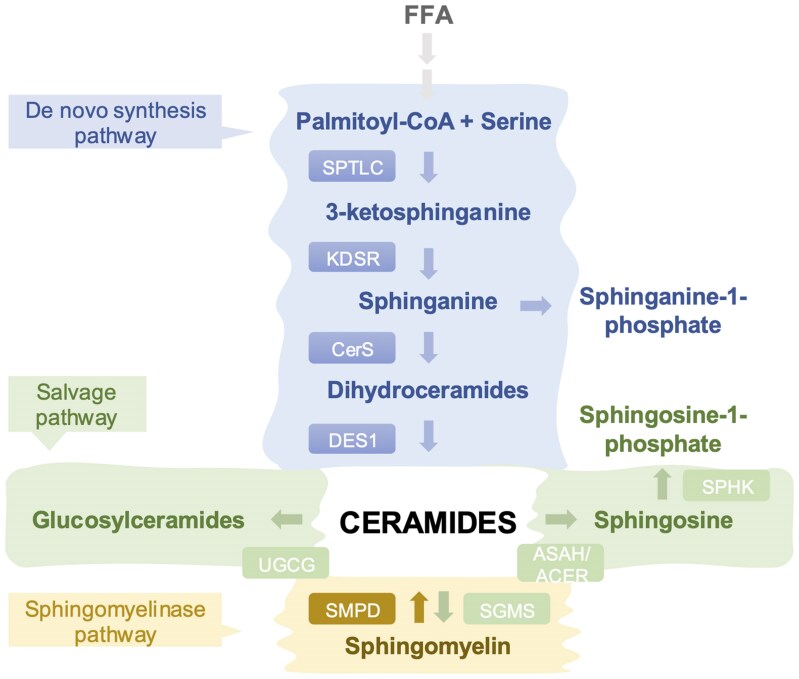
Sphingolipid pathway and metabolism in cardiovascular system.

As previously mentioned, Cer play a fundamental role in the sphingolipid biosynthesis pathway. Two different pathways promote Cer accrual: the *de novo* synthesis pathway and the sphingomyelinase pathway.^35,36^ The *de novo* pathway starts at the endoplasmic reticulum level with the condensation of palmitoyl-CoA and serine by the rate-limiting enzyme serine palmitoyltransferase (SPTLC1, 2, and 3) to form 3-ketosphinganine^37^ (*Figure 1*). The latter is then reduced to form sphinganine by 3-ketosphinganine reductase (KDSR). Next, sphinganine gets modified by the broad class of enzymes, Cer synthases (CerS) (CERS1-CERS6), by adding different acyl chain lengths of FA to form several dihydroceramides that differ based on the number of carbon constituting the acyl chain length.^38^ CERS2, CERS4, and CERS5 are highly expressed in heart tissue and have a strong inter-regulatory effect to compensate for the loss of either isoform.^39^ Dihydroceramide desaturases (DES1) then modifies dihydroceramides to Cer by adding a double bond to the sphingoid backbone.^40^ The second important pathway in acute production of Cer is the sphingomyelinase pathway, catalysed by sphingomyelinases (SMPD1–5) which convert sphingomyelin (SM) (crucial sphingolipid in maintaining cell membrane structure) to Cer. Cer then follow one of the three pathways (catalytic or salvage pathways) to form: (i) SM through the activity of sphingomyelin synthases (SGMS1 and 2),^41^ (ii) sphingosine and sphingosine-1-phosphate (S1P) through the activity of ceramidases (encoded by ASAH1, 2 and ACER1, 2, and 3) and sphingosine kinases (SPHK1 and 2), respectively,^42,43^ or (iii) glucosylceramides through the activity of UDP-glucose ceramide glucosyltransferase (UGCG).^44^ Glucosylceramide is the precursor of the large family of glycosphingolipids, which consist of a Cer backbone connected to a sugar moiety projected into the extracellular space.^45^ Together, these reactions are essential in maintaining the pool of bioactive lipids.^46^

## Association between circulating sphingolipids and heart failure in humans

### Circulating sphingolipids in heart failure with reduced ejection fraction patients

Sphingolipids are transported in the bloodstream through lipoprotein particles, such as VLDLs, LDLs, and HDLs.^47^ The signalling function of circulating sphingolipids and their association with HF remains to be elucidated. Currently, published studies on circulating sphingolipids have examined their potential role as predictive biomarkers in healthy and diseased patients in the context of various diseases such as cancer, obesity, and atherosclerosis.^48–50^ De Carvalho *et al*.^51^ identified 12 plasma Cer as a prognostic signature for predicting long-term major adverse cardiac and cerebral event rates following a myocardial infarction (MI). Similarly, the Mayo Clinic validated these findings in a cohort of healthy individuals to assess the risk of MI and stroke using Cer as lipid biomarkers. Laaksonen *et al*.^52^ demonstrated that the Cer16:0/Cer24:0 ratio had a higher predictive value for cardiovascular death compared to other established scores such as the Global Registry of Acute Coronary Events (GRACE) score and Marschner score. Furthermore, through a machine learning approach, Poss *et al*.^7^ validated serum Cer as candidate biomarkers of cardiovascular disease in 462 healthy patients. The significant predictive value of Cer has led the Mayo Clinic to incorporate the atherosclerotic Cer score in their clinical practice to stratify the risk of adverse cardiovascular events in patients.^8^

In light of the potential of sphingolipids as biomarkers for atherosclerotic diseases, several research groups have focused their studies on the association between circulating sphingolipids and chronic HF. Lemaitre *et al*.^6^ conducted a study in which they measured plasma sphingolipid in a biracial cohort of older adults who were followed up for 9.4 years. Their findings revealed that higher levels of Cer16 and SM16 (containing palmitic acid) were associated with an increased risk of incident HF (HFrEF or HFpEF), whereas lower levels of Cer22 and SM22 (carrying any very long-chain FA) were associated with a lower risk of incident HF. The outcome of Cer16 with HF incidence did not reach significance when examining each of HFrEF and HFpEF independently. In the Framingham Heart Study and the Study of Health in Pomerania cohorts, the Cer24/Cer16 ratio and Cer24 were found to be inversely associated with incident chronic heart disease and incident HF.^53^ Similarly, in a cohort of 400 ambulatory chronic HF patients, patients who died from cardiovascular causes exhibited higher levels of plasma Cer16 and Cer24:1, while Cer22 and Cer24 were lower. The ratio of each Cer with Cer24 was also positively associated with cardiovascular death in the same cohort, even after adjusting for known cardiovascular risk factors such as serum creatinine, medication use, New York Heart Association Functional Classification (NYHA), ischaemic HF aetiology, and left ventricular ejection fraction.^54^ Yu *et al*.^55^ observed a significant stepwise increase in total Cer levels with NYHA functional classes. Lastly, in a cohort of 34 moderate and advanced HF patients, serum levels of Cer16, Cer18, Cer20:1, Cer20, Cer22:1, and Cer24:1 were increased in HF compared to control subjects, and circulating Cer levels remained unchanged following left ventricular assist device (LVAD) unloading in the same cohort.^56^

Furthermore, other sphingolipids have attracted considerable interest in the cardiovascular field, particularly S1P, which has been correlated with both negative and positive cardiovascular outcomes. The S1P binds to three types of receptors that are found on cardiomyocytes: S1PR1, S1PR2, and S1PR3.^57^ The involvement of S1P and S1PRs has shown contrasting effects in the cardiovascular field, primarily in MI, myocarditis, atherosclerosis, and hypertension. Polzin *et al*.^58^ reported lower circulating S1P levels in ischaemic heart disease patients with HFrEF compared to patients with HFpEF. Additionally, plasma S1P appeared to be lower in NYHA Class III and IV patients compared to NYHA Class I and Class II patients. Other studies have identified associations between abnormal plasma S1P levels in patients with chronic systolic HF and cardiovascular mortality.^59^ Conversely, Brakch *et al*.^60^ found that patients with Fabry disease had higher levels of plasma S1P. By supplementing healthy mice with S1P, they were able to induce cardiac hypertrophy and achieve cardiovascular remodelling similar to that observed in patients with Fabry disease.

### Circulating sphingolipids in heart failure with preserved ejection fraction patients

Considering the critical association between obesity, diabetes mellitus, and HFpEF, sphingolipids have been studied in this context as well. Brady *et al*.^61^ performed lipidomics analysis on 49 patients with type 2 diabetes mellitus and correlated their findings with change indices in cardiac remodelling. They found a positive association between left ventricular mass:volume ratio and three SM species [hydroxypalmitoyl SM, SM (d18:2/16:0, d18:1/16:1) and SM (d18:2/24:1, d18:1/24:2)] and four Cer-derived species [glucosylceramide (d18:2/24:1, d18:1/24:2), glycosyl-N-nervonoyl-So (d18:1/24:1), lactosyl-N-nervonoyl-So (d18:1/24:1), and lactosyl-N-palmitoyl-So (d18:1/16:0)], with only one Cer-derived species [lactosyl-N-palmitoyl-So (d18:1/16:0)] being negatively associated with left ventricular filling pressures (E/e′ ratio). In another study performed by O’Sullivan *et al*.,^62^ the group investigated myocardial fuel using simultaneous arterial and coronary sinus sampling in 13 healthy control subjects and 20 patients with HFpEF. With regard to studied lipids, they found that TAG (16:0_14:0_22:6) was significantly extracted by HFpEF myocardium with more lipids showing significance when HFpEF patients were separated by sex. They show that male HFpEF extracted 12 lipids, TAGs (16:0_18:1_22:5; 18:4_18:1_18:1; 29:0_18:2_18:2), and Cer (d19:1_22:0) and released 8 other lipids [phosphatidylinositols (18:0_20:2), hexosylceramide (Hex1Cer) (d18:2_24:0), and TAGs (16:0_16:0_20:3)]. The most common lipid used in male HFpEF was TAGs, followed by lower double bond content of both Hex1Cer and Cer. With the latter study, the group concludes that HFpEF patients preserve a certain level of FA uptake, with differences in lipid uptake seen between males and females. In a study involving 12 patients with obesity-related HFpEF who underwent gastric bypass surgery, plasma lipidomics analysis was performed before the procedure and 3 months after. The authors reported a significant decrease in HF symptoms and NYHA class, along with a regression in cardiac mass 6 months after the surgery. After 3 months of weight loss, 4 Cer and 12 SM showed a significant decrease, although none of them correlated with improvement in diastolic function.^63^

### Limitations and future directions

While associations between circulating sphingolipids and the development and progression of HF have been described, these correlations do not establish causation. Further studies are required to elucidate the direct mechanistic links between circulating sphingolipids and the pathogenesis of HFrEF and HFpEF. Specifically, understanding how elevated levels of different lengths of Cer and S1P influence cardiac tissue—either directly through intracellular signalling or indirectly via systemic biosignalling pathways—remains a critical area of investigation. Given the conflicting roles of S1P and long-chain sphingolipids in cardiovascular health, it is plausible that certain sphingolipid species may exert cardioprotective effects, thereby offering potential therapeutic avenues for targeted intervention. Notably, differences in the circulating sphingolipid profiles between HFrEF and HFpEF suggest distinct pathophysiological mechanisms underlying these two conditions, reinforcing that they should not be managed with a uniform therapeutic approach. It is also crucial to acknowledge that HF is not merely a cardiac disorder but a systemic condition involving multiple organ systems, including the liver, kidneys, and skeletal muscle. These organs, which are known to be affected in sphingolipidoses, likely play an integral role in the systemic regulation of sphingolipid metabolism in HF. However, the interplay between these organ systems and the contributions of sphingolipids to multi-organ dysfunction in HF remain largely unexplored. Future research should aim to elucidate these interconnections, as a more comprehensive understanding of sphingolipid dynamics across organ systems may provide novel insights into disease progression and potential therapeutic strategies.

## Association between myocardial tissue sphingolipids and heart failure in humans

### Sphingolipids in heart failure with reduced ejection fraction patients

Ji *et al*.^56^ conducted an analysis on myocardial Cer in patients with advanced HF who underwent LVAD implantation and transplantation. They observed elevated levels of total Cer, as well as specific subtypes such as Cer16:1, Cer16, and Cer24:1, in the failing myocardium compared to non-diseased hearts. Interestingly, the levels of myocardial Cer were not influenced by cholesterol, LDL, HDL, or TG levels in patients. The study also noted an increase in SPTLC2 in the failing myocardium. Following mechanical unloading through LVAD support, the total levels of myocardial Cer and specific subtypes Cer16:1, Cer16, Cer20:1, Cer20, Cer22:1, and Cer24:1 decreased in patients with advanced HF compared to pre-implantation values. In another study by Pérez-Carillo *et al*., cardiomyocytes from 42 patients with end-stage HF undergoing heart transplantation were examined. The levels of four bioactive sphingolipids (S1P, Cer, sphingosine, and SM) were quantified using enzyme-linked immunosorbent assay (ELISA). Significant alterations in S1P and Cer levels were observed between HF tissue and donor tissue. The study also identified 12 differentially expressed genes involved in sphingolipid biosynthesis and salvage pathways. In the *de novo* pathway, down-regulation of SPTLC1 and SPTLC3 was observed, along with up-regulation of CERS1, which catalyses the production of C18-containing Cer. In the salvage pathway, the expression of SGPP1, SGPP2, S1PR3, and ACER1 was down-regulated while ACER3 was up-regulated. These changes in gene expression of enzymes involved in Cer turnover suggest a potential damaging role in the pathophysiology of HF.^64^ However, whether this dysregulation is a consequence of the failing heart or a contributing factor remains uncertain. With regard to mechanism behind Cer-induced HF phenotype, studies on animal models described below are enlightening.

### Sphingolipids in heart failure with preserved ejection fraction patients

Human biopsies from HFpEF patients are very scarce, and data on the role of sphingolipids on HFpEF remain limited. In the following study from Hahn *et al*.,^65^ myocardial biopsies were taken from right ventricle in 45 patients with HFpEF and were compared to right ventricular tissue from both healthy controls (*n* = 20) and HFrEF patients undergoing cardiac transplantation (*n* = 30). Following principal component analysis, one of the major metabolites separating HFpEF patients from controls and HFrEF patients was medium- and long-chain FA. Moreover, HFpEF myocardium had a reduction in genes involved in FA uptake and oxidation compared to controls. Further research and clinical studies are needed to better understand the role of sphingolipids in HFpEF.

### Limitations and future directions

At the tissue level, most studies to date have been conducted with small patient cohorts, limiting their statistical power and generalizability. To better delineate the role of sphingolipids and their associated enzymatic pathways in HF pathophysiology, larger and more comprehensive studies—particularly longitudinal investigations—are essential. These studies should aim to clarify the contribution of sphingolipids to myocardial bioenergetics and metabolic adaptations in both HFrEF and HFpEF. Animal models, particularly murine models, provide a valuable platform for these investigations and should be leveraged to bridge the translational gap to human pathology. Moreover, disparities in study design, including single-centre vs. multi-centre trials, may introduce variability due to differences in clinical protocols, patient demographics, and environmental influences. Standardization issues in blood and tissue collection, processing, and storage conditions also play a crucial role, as variations in sample handling can significantly impact sphingolipid stability and composition. Furthermore, technological advancements in lipidomics, particularly in mass spectrometry-based methodologies, have evolved over time, leading to potential discrepancies in data interpretation between older and more recent studies. Differences in analytical techniques, data normalization approaches, and bioinformatics pipelines may further contribute to the observed inconsistencies.

## Sphingolipids and heart failure in murine models

### Fat and sphingolipid metabolism in genetically modified rodent models

The role of fat in the development of heart disease is multifactorial and complex. It serves as the primary source of ATP, essential for energy expenditure, while also contributing to lipotoxicity and playing a role in the pathogenesis of HF. Yagyu *et al*.^66^ created a heart-specific lipoprotein lipase (hLpL^GPI^) transgenic mice model which led to increased entry of fat in cardiomyocytes. This animal model developed HFrEF characterized by left ventricular dilation and reduced ejection fraction. Histologically, hLpL^GPI^ hearts showed accumulation of neutral lipids in cardiomyocytes, a disarrayed architecture, and increased mitochondria but no change in levels of TAG in cardiomyocytes. Cardiac lipidomics performed on this model also reveals accumulation of cardiac Cer. Treatment with an inhibitor of *de novo* sphingolipid biosynthesis decreased the production of Cer and ameliorated the phenotype. Similarly, the overexpression of a different lipid transporter led to the excessive entry and storage of FA in cardiomyocytes and the development of HF. The cardiomyocyte-specific transgenic overexpression of FATP1 in murine led to the increased entry of fat into cardiomyocytes and the development of HFpEF phenotype,^67^ while cardiac-specific overexpression of acyl-CoA synthase 1 also resulted in cardiomyocyte lipid accumulation, and eventually the development of HFrEF phenotype.^68^ Given these recent findings, it remains unclear which mechanisms or defects would drive the development of HF with reduced or preserved ejection fraction, as the end point of cardiomyocyte lipid accumulation has been shown to lead to both phenotypes. To further investigate the role of sphingolipids and Cer in the development of HF, cardiomyocyte-specific genetic models are described in *Table 2*.

**Table 2:** Cardiac phenotype of genetically engineered mice with enzyme modifications in the sphingolipid pathway

Enzyme	Experimental model	Cardiac phenotype	Circulating and cardiac tissue lipidomics	Reference
SPTLC1	Whole-body heterozygous LCB1 KO crossed with LpL^GPI^	Improved HFrEF compared to LpL^GPI^ KO model	Reduced cardiac Cer compared to WT	^90^
SPTLC2	MHC-Cre/Sptlc2^loxP/loxP^	Dilated cardiomyopathy and HFrEF	35% reduction in cardiac Cer compared to WT, with major reduction in Cer18, Cer20, Cer24, and Cer24:1. Decrease in dihydroceramides and sphingosine. No change in S1P, SM, Cer14, and Cer16	^35^
SMPD1	Whole-body Smpd1^−/−^ KO	N/A	Reduced cardiac Cer compared to WT	^105^
ASAH1	Whole-body *Asah1*^P361R/P361R^ mice	N/A	Increased cardiac Cer compared to WT	^106^
UGCG	Cardiomyocyte-specific UGCG knockdown hUGCG^−/−^	Dilated cardiomyopathy and HFrEF	Reduction in lactosylCer in cardiac tissue and both glucosylCer and lactosylCer levels reduced in cardiomyocytes compared to WT	^21^

MHC, myosin heavy chain; KO, knockout; WT, wild-type.

### Heart failure with reduced ejection fraction rodent model

Heart failure with reduced ejection fraction is a heterogeneous syndrome which can arise from various aetiologies and pathophysiological mechanisms. To better understand the complexity of HF and develop effective therapeutic strategies, several murine models have been established to mimic distinct aspects of the human disease. These models enable the investigation of specific pathogenic pathways and the evaluation of potential interventions. Among the commonly utilized murine models of HF are those induced by MI through left anterior descending (LAD) artery ligation, pressure overload induced by transverse aortic constriction (TAC) and drug-induced modelling a hypertensive model.^69^ In the following section, we reviewed the most frequently employed murine models of HF and assess their impact on cardiac and circulating lipidome profiles.

#### Myocardial infarct by left anterior descending ligation

Ji *et al*.^56^ induced MI by LAD artery ligation in mice and conducted follow-up examinations at 2 and 10 weeks post-procedure to assess changes in Cer levels. Two weeks after the procedure, elevated circulating free FA, total cholesterol, and total circulating Cer levels, including Cer16, Cer18, Cer24:1, Cer24, and Cer26:1, were observed, along with increased total myocardial Cer levels, including Cer14, Cer18, Cer20:1, Cer20, and Cer22:1. At 10-week time point, cholesterol levels returned to baseline, while free FA and TAG remained elevated. Circulating Cer levels were similar between sham-operated and post-MI mice, whereas myocardial total Cer levels, particularly Cer16, Cer24:1, and Cer24, were significantly increased at 10 weeks compared to sham-operated controls. The authors concluded that up-regulation of SPTLC2 expression in this mouse model contributed to the observed lipid alterations. Additionally, pharmacological inhibition of the *de novo* Cer synthesis pathway using myriocin rescued the HF phenotype following MI. These findings were validated in an SPTLC2-knockout model, wherein knockout mice exhibited preserved cardiac function following LAD ligation compared to wild-type counterparts.

#### Transverse aortic constriction

In the TAC model, sphingolipid levels were assessed in heart tissue samples at three time points, 1 day, 1 week, and 8 weeks post-TAC, and compared to sham-operated controls. Analysis revealed no significant changes in sphingolipid levels 1 day after the procedure compared to sham-operated controls. However, at the 1-week and 8-week time points, there was a notable reduction in total myocardial levels of erythro-sphingosylphosphorylcholine, sphingosine, stearoyl SM, and total dihydrosphingosine compared to the control group. The observed depletion in sphingolipids may result from either increased utilization or decreased synthesis, as speculated by the authors.^70^ Similarly, in an another study, mice subjected to TAC were monitored for 14 weeks, after which myocardial lipidomic analysis was conducted. The results revealed a significant increase in myocardial total Cer levels, particularly Cer16, Cer24:1, and Cer24.^71^

#### Hypertension-induced heart failure

The induction of HF in mice through the administration of drugs such as isoproterenol, phenylephrine, or angiotensin II can stimulate a hypertensive aetiology of HF. Despite the significance of this model, there is a notable gap in the literature regarding the characterization of circulating and myocardial lipidomic profiles in mice solely subjected to drug-induced HF. However, RNA sequencing analyses conducted on murine hearts following isoproterenol-induced HF have revealed a down-regulation of genes associated with FA oxidation pathways.^72^

### Heart failure with preserved ejection fraction rodent model

Heart failure with preserved ejection fraction remains a multifaceted diagnostic challenge, characterized by a paucity of comprehensive understanding. As currently described, HFpEF more commonly develops with older age and is more likely to affect women, with comorbidities such as obesity, diabetes, and hypertension. Given the unresolved intricacies surrounding its pathogenesis and diagnostic criteria, the scientific community faces considerable challenges at developing an ideal animal model for HFpEF. Consequently, we will discuss the different HFpEF models below, with the future hope of elucidating the various pathways culminating in the development of HFpEF as currently recognized.

#### Single-hit model

##### External interventions

###### High-fat diet

Two different types of diet have been studied in the development of HFpEF: milk fat-based diet^73,74^ and high-fat diet (HFD).^75^ In both cases, mice develop profound insulin resistance and impaired glucose tolerance that accompanies an obese phenotype. Concentric cardiac hypertrophy and reduced cardiac function were observed after 18 weeks on MFBD, accompanied by increased total myocardial d18-Cer and d16-Cer, Cer14 (at 8 weeks and 16 weeks on milk fat-based diet), Cer18, Cer18:1, Cer22, Cer24, Cer d16:1/C18:0, Cer d16:1/C20:0, Cer d16:1/C22:0, and Cer d16:1/C24:0 (at 18 weeks on milk fat-based diet) compared to chow-fed controls.^73^ After 10 weeks on HFD, no HFD-induced cardiac dysfunction was observed although the effect of insulin resistance and mitochondrial oxidation was significantly different at this time point compared to low-fat diet controls. No change in myocardial Cer was observed in mice on HFD at 10 weeks.^75^

##### Genetically modified models

###### Obesity and diabetes

When using the genetically modified diabetes model db/db (leptin receptor mutant), diastolic dysfunction was seen at 15 weeks of age but not at 12 weeks of age. Total myocardial Cer including Cer16, Cer18, Cer18:1, Cer20, Cer22:1, Cer23, and Cer24:1 were increased at week 12 while total myocardial Cer including Cer16, Cer18, Cer18:1, and Cer23 were decreased at week 15 compared to their respective wild type.^76^ Cardiac function has not been studied in the ob/ob mouse model, which congenitally lacks leptin expression, but Raichur *et al*.^77^ observed variations between the ob/ob plasma Cer profiling and control mice.

#### Two-hit model

##### Hypertension ± high-fat diet

To simulate a hypertensive model, Pellieux *et al*.^78^ explored the use of cardiomyocyte-specific angiotensin II overexpression mice. Cardiac hypertrophy was observed in all transgenic mice, but a decrease in cardiac power was observed in only 51% of transgenic mice fed a standard diet. Following an 8-week duration of HFD, the transgenic group with lower cardiac power observed a further reduction of cardiac power compared with standard chow-fed control. The latter findings were also accompanied by increased myocardial Cer compared to controls. There was no change in myocardial Cer between transgenic mice and control when on standard diet.

#### Three-hit model

Considering the significance of comorbidities in patients with HFpEF development, employing a murine three-hit model would provide the most accurate simulation of HFpEF pathogenesis in humans. Deng *et al*.^79^ developed a model integrating age, hypertension, and obesity by subjecting 3-month old mice to a HFD for 13 months, along with desoxycorticosterone pivalate injections during the final month. Mice subjected to this three-hit treatment exhibited pathological cardiac hypertrophy alongside with preserved ejection fraction but a decrease in cardiac output compared to 16-month-old controls. Furthermore, these observations were associated with a decrease in cardiomyocyte FA uptake. However, investigation into the downstream sphingolipid pathway was not conducted.

### Future directions

In murine models, lipid accumulation within the myocardium has been implicated in the development of both HFrEF and HFpEF. However, a deeper understanding of the mechanistic underpinnings of these processes is necessary to distinguish the differential effects of specific lipid species. Additionally, discrepancies in findings between HFrEF and HFpEF models furthermore underpin the heterogeneity of HF. Future studies should employ advanced lipidomics techniques to characterize the specific types of lipids that accumulate in cardiac tissue and investigate their functional consequences on myocardial metabolism and contractility. Despite similarities in sphingolipid profiles between HFrEF and HFpEF, the precise mechanistic roles of sphingolipids in disease progression remain poorly understood. Given the extensive biosignalling roles of sphingolipids in various tissues, their implications for cardiac function require more in-depth exploration.

## Conclusion and future directions

In this review, we aim to synthesize recent advancements linking sphingolipid metabolism to the pathophysiology of HF. While substantial progress has been made in elucidating the roles of Cer and S1P in HF development and progression, the functions of other sphingolipid species remain poorly understood. A critical aspect of this research is the translation of findings from murine models to human pathophysiology, with the ultimate goal of identifying specific bioactive sphingolipids and their downstream signalling pathways as potential therapeutic targets for HF.

In the recent elucidation of HF pathophysiology, particularly in the context of HFrEF, several institutions have highlighted the potential for failing hearts to recover both function and structure despite prior insults.^80,81^ The optimal achievement of myocardial recovery necessitates tailored interventions, contingent upon precise patient selection and management strategies.^82–85^ At the biological level, various pathways contribute to the phenomenon of recovery, including but not limited to t-tubules and glycolytic pathways.^28,86–88^ However, the specific role of FA, sphingolipids, and Cer in the recovery process remains to be determined. Interestingly, inhibitors targeting the *de novo* pathway and Cer production, such as myriocin, have exhibited the potential to rescue the HF phenotype in murine models.^89,90^ Given the uncertain contributions of other sphingolipids in the development of HF, the exploration of additional targets and modulators within the sphingolipid pathway represents a compelling direction for investigating HFrEF and HFpEF treatments.

## Data Availability

No new data were generated or analysed in support of this research.

## Lead author biography

Dr Tseliou is a principal investigator leading a laboratory focused on the role of fatty acid metabolism in cardiovascular diseases. Her current NIH-funded research explores how sphingolipids influence myocardial recovery, using tissue and single-cell omics to study cardiomyocyte metabolism. Her team works with mouse models and engineered heart tissue to identify mechanisms that contribute to adult cardiomyopathy and aims to develop precision therapeutics. Additionally, Dr Tseliou is a board-certified cardiologist with expertise in advanced heart failure and cardiac transplantation.
